# Longitudinal impact of dapagliflozin treatment on ventricular repolarization heterogeneity in patients with type 2 diabetes

**DOI:** 10.1111/jdi.13063

**Published:** 2019-05-15

**Authors:** Tatsuya Sato, Takayuki Miki, Shinya Furukawa, Bunzo Matsuura, Yoichi Hiasa, Hirofumi Ohnishi, Masaya Tanno, Tetsuji Miura

**Affiliations:** ^1^ Department of Cardiovascular, Renal and Metabolic Medicine Sapporo Medical University School of Medicine Sapporo Japan; ^2^ Department of Cellular Physiology and Signal Transduction Sapporo Medical University School of Medicine Sapporo Japan; ^3^ Department of Epidemiology and Preventive Medicine Ehime University Graduate School of Medicine Toon Ehime Japan; ^4^ Department of Lifestyle‐related Medicine and Endocrinology Ehime University Graduate School of Medicine Toon Ehime Japan; ^5^ Department of Gastroenterology and Metabology Ehime University Graduate School of Medicine Toon Ehime, Japan; ^6^ Department of Public Health Sapporo Medical University School of Medicine Sapporo Japan

## Abstract

QTc dispersion (QTcd) tended to be decreased at 24 weeks and was significantly decreased at 2 years after dapagliflozin treatment. In the subgroup with QTcd?53.7 ms (median), QTcd was significantly decreased at 24 weeks and remained improved for 2 years. Dapagliflozin also significantly reduced Tpeak‐Tend/QT in a subgroup with Tpeak‐Tend/QT?0.25 (median).

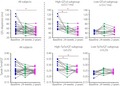

Increased ventricular repolarization heterogeneity (VRH) is one of the predictors of poor cardiovascular prognosis in patients with type 2 diabetes. Our retrospective studies^1^, ^2^ have shown that increased VRH in patients with type 2 diabetes is reversed by treatment with a sodium–glucose cotransporter 2 (SGLT2) inhibitor, but not by other oral hypoglycemic agents, and the result of a recent retrospective study^3^ also suggested that an SGLT2 inhibitor has a beneficial effect on VRH in type 2 diabetes. However, to our knowledge, no study in the literature has prospectively examined the effect of an SGLT2 inhibitor on type 2 diabetes‐induced VRH.

Thus, we prospectively assessed QTc dispersion (QTcd) and Tpeak‐Tend/QT in lead V5, electrocardiographic indices of VRH, in 25 patients with type 2 diabetes who had been treated with dapagliflozin 5 mg once daily for 2 years. This study was approved by the institutional review board of Ehime University Graduate School of Medicine. Inclusion and exclusion criteria were as described previously^2^, ^4^. Baseline patient characteristics were as follows: 60% participants were men, age was 57.8 ± 12.2 years, median duration of diabetes was 13.0 years (interquartile range [IQR] 7.3–20.5 years), body mass index was 30.0 ± 9.2 kg/m^2^, glycated hemoglobin was 7.6 ± 1.1% and blood pressure was 139 ± 18/80 ±14 mmHg. At 24 weeks after the treatment, body mass index, glycated hemoglobin and blood pressure were decreased to 27.4 ± 4.1 kg/m^2^, 7.2 ± 0.8% and 127 ± 13/70 ± 11 mmHg, respectively, and the trends were maintained thereafter.

QTc dispersion tended to be decreased at 24 weeks, and was significantly decreased at 2 years after dapagliflozin treatment compared with that at baseline (Figure 1). As the protective effect of SGLT2 inhibitors was greater in patients with larger VRH at baseline^2^, ^3^, the patients were divided into two subgroups by the median of QTcd (53.7 ms). In the subgroup with QTcd ≥53.7 ms, QTcd was significantly decreased at 24 weeks (from 68.3 ms [IQR 57.0–83.6 ms] at baseline to 56.5 ms [IQR 45.3–61.6 ms], *P* = 0.022), and remained improved for 2 years (46.1 ms [IQR 39.9–58.3 ms], *P* = 0.002). In contrast, in the subgroup with QTcd <53.7 ms, QTcd (48.6 ms [IQR 42.4–51.7 ms] at baseline) was unaffected by treatment. As for Tpeak‐Tend/QT, improvement by dapagliflozin was not significant when all participants were included in the analysis. However, in a subgroup with Tpeak‐Tend/QT ≥0.25 (median), dapagliflozin significantly reduced Tpeak‐Tend/QT at 24 weeks (from 0.267 [IQR 0.254–0.323] at baseline to 0.262 [IQR 0.243–0.274], *P* = 0.008) and at 2 years (0.255 [IQR 0.243–0.265], *P* =0.013). Changes in QTcd and Tpeak‐Tend/QT were not correlated with change in glycated hemoglobin after dapagliflozin treatment, even in the larger VRH subgroups. Heart rate and QTc interval were not altered by dapagliflozin treatment.

**Figure 1:**
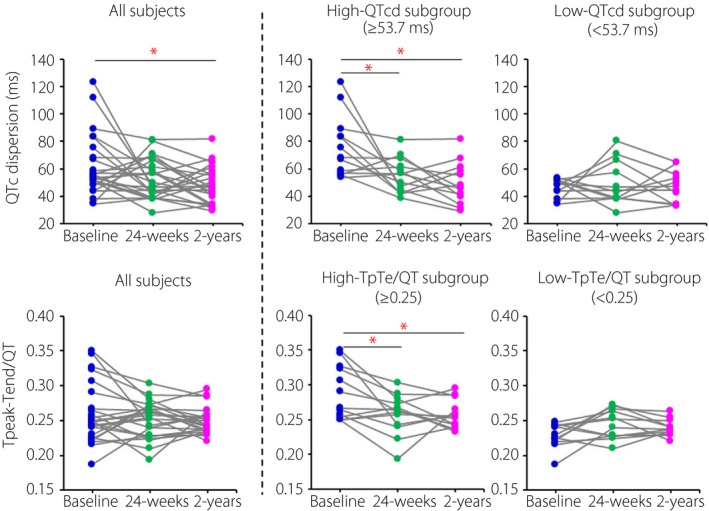
Upper: QTc dispersion (QTcd) before and at 24 weeks and 2 years after the start of dapagliflozin treatment in all patients, in a high‐QTcd subgroup (QTcd ≥53.7 ms) and in a low‐QTcd subgroup (QTcd <53.7 ms). Lower: Tpeak‐Tend/QT ratio (TpTe/QT) before and at 24 weeks and 2 years after dapagliflozin treatment in all patients, in a high‐TpTe/QT subgroup (TpTe/QT≥ 0.25) and in a low‐TpTe/QT subgroup (TpTe/QT<0.25). Comparison of the repeated measures using Friedman's test and additional post‐hoc analysis with Wilcoxon's signed‐rank test was carried out. *P < 0.05.

Together with retrospective studies^2^, ^3^, the present prospective study supports the notion that treatment with an SGLT2 inhibitor improves VRH in patients with type 2 diabetes for years. Intriguingly, the improvement of VRH after SGLT2 inhibitor treatment was independent of glycemic control, as was found in a previous study^2^, and thus it might be attributable to pleiotropic effects of this class of agents. Nevertheless, as VRH is a risk factor of lethal cardiac events, reversal of diabetes‐induced VRH is one of the possible mechanisms by which SGLT2 inhibitors reduce cardiac mortality, particularly sudden cardiac death, in patients with type 2 diabetes.

## Disclosure

TS, TaM, SF, MT and TeM received lecture honoraria for lectures from Ono Pharmaceutical Co., Ltd. and AstraZeneca. The other authors declare no conflict of interest.
